# Deep Q-Learning for Two-Hop Communications of Drone Base Stations

**DOI:** 10.3390/s21061960

**Published:** 2021-03-11

**Authors:** Azade Fotouhi, Ming Ding, Mahbub Hassan

**Affiliations:** 1 Research and Innovation Department, Altran Technologies, 78140 Velizy-Villacoublay, France; 2 Data61, CSIRO, Sydney 2015, Australia; ming.ding@data61.csiro.au; 3 School of Computer Science and Engineering, University of New South Wales (UNSW), Sydney 2052, Australia; mahbub.hassan@unsw.edu.au

**Keywords:** drone base station, deep Q-learning, Q-learning, autonomous navigation, UAV

## Abstract

In this paper, we address the application of the flying Drone Base Stations (DBS) in order to improve the network performance. Given the high degrees of freedom of a DBS, it can change its position and adapt its trajectory according to the users movements and the target environment. A two-hop communication model, between an end-user and a macrocell through a DBS, is studied in this work. We propose Q-learning and Deep Q-learning based solutions to optimize the drone’s trajectory. Simulation results show that, by employing our proposed models, the drone can autonomously fly and adapts its mobility according to the users’ movements. Additionally, the Deep Q-learning model outperforms the Q-learning model and can be applied in more complex environments.

## 1. Introduction

Recent advancements in Unmanned Ariel Vehicles (UAVs) or Drones, along with the development of communication technology, have changed and led to the emergence of various civilian applications such as emergency medical equipment deliveries [1], real-time monitoring in mining environmental [2], precision agriculture and crop monitoring [3] and traffic monitoring in smart cities [4]. Moreover, one of the most important applications of drones is in cellular networks, where the drones can act as a flying base station to enhance coverage and improve the quality of communication services for the ground users.

Despite all the advantages that can be brought into a cellular network by drones, they still face many challenges for integrating and operating in networks. Both research and industrial communities showed interest in working on overcoming these challenges. Multiple study items on the integration of UAVs into future cellular networks have been released recently by the 3rd Generation Partnership Project (3GPP) [5,6]. Various features of integration of drones such as deployment, trajectory planing, power consumption, security, and so forth are studied by many researchers as well ([7,8,9]).

One of the main important challenges in the application of drones is providing backhaul link and ensuring its performance. The backhaul link is established between a drone base station (DBS) and a macrocell BS, where the the access link is between a user and the DBS. Although the efficiency of the backhaul link must be considered in the drone deployments, most of the existing works ignored this issue and have only focused on the access link performance. That is why, in this work, we study a two-hop communication model (backhaul link and access link) to improve the system’s performance. We consider a practical scenario where a flying DBS is serving mobile users in the environment through a two-hop communication model. Q-learning and Deep Q-learning based algorithms are proposed to optimize the navigation of the drone. The performance of the proposed algorithms is evaluated in different network conditions. Additionally, a simple algorithm that targets maximizing the access link capacity is studied in order to point out the importance of the proposed algorithms and the two-hop communication model.

In our previous work [10], we modeled the network as a simple reinforcement learning (RL) problem, in which each drone base station tries to learn its navigation assuming that there is a high probability for the other agents to follow their previous direction. In this work, we have considered a complex model, where the drone learns its trajectory based on Q-leaning (QL) and Deep Q-learning network (DQN), taking into consideration the mobility of the ground user. Our contributions are summarized as follows:

- Considering two-hop communication ( i.e., the access link and the backhaul link) to optimize the drone base station trajectory and to improve network performance,
- Designing Q-learning and Deep Q-learning models to solve the joint two-hop communication scenario.
- Taking into account the mobility of the ground user

The rest of the paper is structured as follows—Section 2 reviews the related work, and Section 3 presents the system model and the problem formulation. Then the Q-learning and Deep Q-learning concepts are briefly described in Section 4. Section 5 introduces the proposed drone mobility algorithms. Section 6 presents the performance evaluation and simulation results, and the conclusion is provided in Section 7.

## 2. Related Work

Reinforcement learning is widely used in UAV communication and networking, which helps UAVs to make intelligent local and autonomous decisions [11,12,13]. Various problems such as connectivity maintenance [14], traffic routing [15], data collection [16] and caching and offloading [17] employed reinforcement learning to improve the efficiency and the autonomy of the networks. In this section, we review existing RL and Deep-RL related works on drones in more detail.

A Deep Q-learning model is used in [18,19] where UAVs are employed to remotely charge up Internet of Things (IoT) nodes through Wireless Power Transfer (WPT) and collect their data. The UAV has no information on the battery level and data length of IoT nodes. In order to minimize the data packet loss of IoT nodes DQN is used. The UAV flies over a predefined trajectory, and at each state the UAV chooses an IoT node to transfer power and collect data. In the proposed DQN model, each state is composed of battery levels, data length and the location of UAV (the UAV altitude is constant). The model is proposed with random node selection, and greedy node selection, where the results clearly show that DQN model reduces the packet loss rate significantly.

Tang et al. [20] has proposed a multi agent deep Q-learning (DQL) model where UAVs are providing energy supply to IoT devices. The static IoT devices are forming disjoint clusters, each of them is served by a UAV that tried to maximize throughput by joint optimization of UAV trajectory and time resource assignment. In the investigated multi agent model, each UAV follows an independent DQL model, while considering the other UAVs as a part of the environment. The simulations indicate that the results can be improved significantly compared to the traditional models. Deep Q learning is also studied in [21] where one UAV aimed to optimize its trajectory in order to maximize the number of served users and the uplink data-rate in emergency situations. According to the simulation results, the proposed DQN model achieves a considerable performance in terms of average coverage over the baseline algorithms such as random and maximum rate. A handover mechanism is developed in [22] using Q-learning to ensure wireless connectivity for the drone-UEs, which are served by terrestrial BSs. A 2D trajectory at fixed altitudes, and predefined potential handover points are considered for the drone-UEs. Each state is composed of the location of the drone, its moving directions and its serving cell. The action to take is to choose one of the k strongest BSs. The compared results with the greedy handover selection show that in the proposed model, the number of handovers is reduced significantly while a reliable connectivity is maintained.

The authors of [23] addressed the UAV trajectory optimization using deep Q-learning where the UAV provides communication services to ground users with the aid of landing spots. The UAV can land on landing spots for saving energy while it continues to serve the users. When the mission is finished, the UAVs is supposed to be at the final position. The ground users are fixed, and the goal is to maximize the sum of the information rate over the whole flying time. Using a neural network, the parameters of the Q-learning are found and the training process is improved. The simulation environment includes 10 users, 2 landing spots and eight movement direction for the UAV.

Similar to our problem, ref. [24] has proposed an RL model to learn the trajectory of a flying base station to serve the ground users. The goal is to maximize the sum rate of transmission during the flying time. The UAV is moving at a constant speed and over a fixed altitude. Unlike our problem, the users are located at fixed locations and the focus is only on the access link.

While most existing works have investigated UAV navigation in a static environment, the authors of [25] addressed a new challenge. They proposed a Double DQL model for UAV navigation to avoid obstacles in a dynamic environment. The states are represented by the image of the environments composed of raw pixel values, and the 8 moving directions at a fixed altitude and speed defined the action space. A DQN model is proposed for UAV navigation through the massive MIMO in [26]. In this model, there exist multiple UAVs that can fly in four different directions. The results show the out-performance of the proposed model over other strategies in terms of coverage and convergence. A Q-leaning based model is proposed in [27] for the intelligent navigation of a UAV, which is equipped with a wireless power transmitter. The goal is to maximize the transmission energy efficiency to the receiving nodes. The transmitter UAV has nine different actions to choose, and the locations of the receiving nodes are known in advance. The results are compared with random movement and static hovering at the center of the environment. The authors of [28,29] used Q-learning to learn UAVs to navigate in an unknown environment to go from an arbitrary location to a target position in the shortest possible way. The results are verified using simulation and real implementation. To guarantee the convergence of the algorithms and to reduce the number of states, the environment is presented by a finite set of circles with equal radius, and the UAV altitude is constant. At each state, the UAV can take four possible actions.

Given that providing backhaul is one of the main challenges in employing drone networks [30,31], this problem is ignored in the presented works. The authors focused more on improving the performance of an access link between the users and the flying base stations. There are few works that consider the two-hop communication such as [32], where the coverage of a typical user in a UAV network is optimized considering both the backhaul link and the end-user link performance. In this work, the UAV adjusts its heights in order to improve the probability of coverage. Dual-hop or multiple hop communication in a UAV relaying system is addressed in [33,34]. The average end-to-end throughput between a pair of fixed transmitter and receiver with the impact of interference in the environment are optimized in these works.

## 3. System Model

We assume that there is a closed square form environment. The Drone Base Station (DBS) starts above an arbitrary location in the environment to provide services to the ground user. A 2D mobility model is considered for the drone where it is flying around at a fixed altitude, $h_{d}$, and a constant speed. The drone moves continuously, as a result, the proposed model can be applied to both fixed-wing and rotary drones. The fixed-wing drones move continuously and can not hold themselves up in the air, while the rotary ones are able to stop flying, stand still, and start to fly again. The user is moving in the target environment according to a mobility model. The user also follows a full buffer traffic model with continuous downloads.

**Figure 1:**
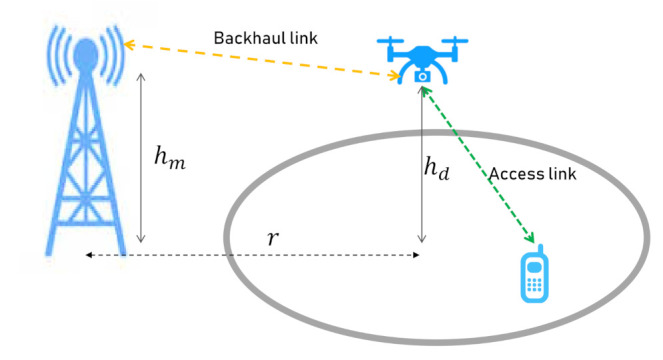
A two-hop communication system model.

A Macro Base Station (MBS) is placed out of our considered environment at height $h_{m}$, and distance *r* from the center of the service area. The MBS is only for backhaul purposes and the communication between the user and the DBS will be offloaded to the MBS. The system model is illustrated in Figure 1.

Moreover, we suppose that the MBS transmits using a fixed transmission power of $p_{m}$, with a frequency of $f_{m}$. The DBS also communicates with a power of $p_{d}$ at frequency of $f_{d}$.

The DBS aims to improve the network capacity for the mobile user taking into account the two-hop communication. Therefore, the DBS needs to adapt its trajectory autonomously according to the movements of the user and the location of MBS, in order to achieve its goal. In this paper, we propose Reinforcement Learning based models for the drone to learn its trajectory by finding a heading direction at each time step.

Following Shannon theorem, the capacity (in bits/sec/Hz) of each link in our two-hop model is given by:

(1) $$\begin{array}{c} C_{d,u}=\log_{2}\left(1+\Gamma_{d,u}\right) \end{array}$$

(2) $$\begin{array}{c} C_{m,d}=\log_{2}\left(1+\Gamma_{m,d}\right) \end{array}$$

where $C_{d,u}$ and $C_{m,d}$ represent the capacity of the access link and the backhaul link, respectively. The signal to noise ratio (SNR) of a transmitter and receiver would be as follows:

(3) $$\begin{array}{c} \Gamma_{d,u}=\frac{p_{d}\times pl_{d,u}}{N_{0}} \end{array}$$

(4) $$\begin{array}{c} \Gamma_{m,d}=\frac{p_{m}\times pl_{m,d}}{N_{0}}, \end{array}$$

where pl is the path loss between the transmitter and the receiver and $N_{0}$ is the power of the additive white Gaussian noise.

We divide the whole operation time into *T* sequential equal time slots, where the overall two-hop capacity that can be delivered to the user at time slot t∈[1,…,T] is defined as the minimum of the access link and backhaul link capacity, as follows:

(5) $$\begin{array}{c} C^{t}=\min(C_{d,u}^{t},C_{m,d}^{t});\;t\in \left[1,\ldots ,T\right]. \end{array}$$

The drone must find a heading direction at each time slot in order to optimize the total two-hop capacity during its mission, hence, the problem is formulated as follows:

(6) $$\begin{array}{c} \begin{array}{cc} A^{*} & ={\mathrm{argmax}}_{\alpha^{t}}\;\frac{\sum_{T}^{t=0}\left(\min(C_{d,u}^{t},C_{m,d}^{t})\right)}{T}, \end{array} \end{array}$$

where $A^{*}=\left\{{\alpha_{1}}^{*},\ldots ,{\alpha_{T}}^{*}\right\},t\in \left[1,\ldots ,T\right]$, is the set of drone’s heading direction during the operation.

We assume that the drone knows its position and the position of the user at any moment.

## 4. Background

In this section, we provide a brief background on Q-learning and Deep Q-learning.

### 4.1. Q-Learning

In Reinforcement Learning (RL), the problem is modeled by a Markov Decision Process (MDP). A MDP is described by a tuple $\left(S,A,P_{a},R_{a}\right)$ where

- *S* is a set of states called the state space,
- *A* is a set of actions called the action space,
- $P_{a}\left(s,s^{\prime }\right)$ is the probability that taking the action a∈A in state s∈S leads to state $s^{\prime }\in S$,
- and $R_{a}\left(s,s^{\prime }\right)$ gives the immediate reward received after transitioning from state *s* to state $s^{\prime }$, due to action *a*.

Q-learning is a model-free RL algorithm where the goal is to learn a policy telling an agent what action to take in a given state. In this model, there is a Q-value for every particular state-action combination, and those Q-values are stored in a Q-table. A higher Q-value represents the higher chance of getting a greater reward.

We denote the Q-value of getting an action *a* in state *s* for policy π as $Q^{\pi }\left(s,a\right)$. Q-values are initialized to an arbitrary value, and in an iterative process, the agent takes different actions and updates the Q-values in the Q-table. At each time step *t*, by performing the action *a* in state *s*, its Q-values are updated according to the following Bellman equation:

(7) $$Q_{t+1}\left(s,a\right)=\left(1-\alpha \right)Q_{t}\left(s,a\right)+\alpha \left[R\left(s,a\right)+\lambda \max_{a^{\prime }\in A}Q_{t}\left(s^{\prime },a^{\prime }\right)\right],$$

where α is the learning rate (0<α≤1), λ is the discount factor (0≤λ≤1) that shows the importance of future reward, and *R* is the immediate reward of this transition.

Because of the exploration/exploitation trade-off, an ϵ-greedy model is used during training and action selection. Instead of always choosing the best learned Q-value action (exploitation), a random action is selected with ϵ probability (exploration). This behavior leads in making random decisions sometimes instead of always selecting the best learned Q-value action. Therefore, for choosing action *a* at state *s* the following model is used:

(8) $$a=\{\begin{array}{cc} \;\mathrm{random}\;\mathrm{action}\in A, & \epsilon \;-probability\\ {\mathrm{argmax}}_{a}\left(Q\left(s\right)\right) & \mathrm{otherwise} \end{array}$$

Eventually, the Q-values converge and the learned values can be used as an action-value function for the agent. However, to learn better and converge faster, Decaying Epsilon Greedy can be used, which tries to decrease the percentage dedicated for exploration over time. At the beginning, the epsilon value is high (close to 1) so that the model can explore and learn things. As it learns, epsilon should decay so that the model can exploit the higher Q-values that are obtained.

The optimal policy is composed of the actions with the highest Q-value for each state, denoted by $Q^{*}\left(s,a\right)$.

### 4.2. Deep Q-Learning

It can be seen that in large or continuous state and action spaces, the size of a Q-table would grow exponentially. Therefore, the Q-table is not practical anymore and a multi-layer neural network (NN) is used to learn and approximate the Q-values. In Deep Q-learning (DQN) the Q-function is approximated to the optimal Q-value by updating the parameter θ or the weights. For a policy π we will have:

(9) $$Q^{\pi }\left(s,a;\theta \right)≅Q^{*}\left(s,a\right).$$

The NN is able to learn efficiently from a few samples and to generalize the model to the whole problem states.

Although DQN is more flexible and practical than Q-learning, there are situations where the network weights can oscillate or diverge, making DQN unstable. One technique to remove correlation and non-stability of DQN in the training process, is “experience replay”. In experience replay, the agent’s experiences are gradually stored in the form of $e=(s_{t};a_{t};r_{t};s_{t+1})$ in a fixed data-set size. During the training, a uniform batch of random experiences are then chosen from this data-set and new experiences are replaced the old ones.

Additionally, two different neural networks to train are used in DQN, which are called “target” and “local” networks. They both start with the same parameters; however, the local network is updated at each time step, while the target network is only updated at scheduled intervals by the parameters of the local network.

**Figure 2:**
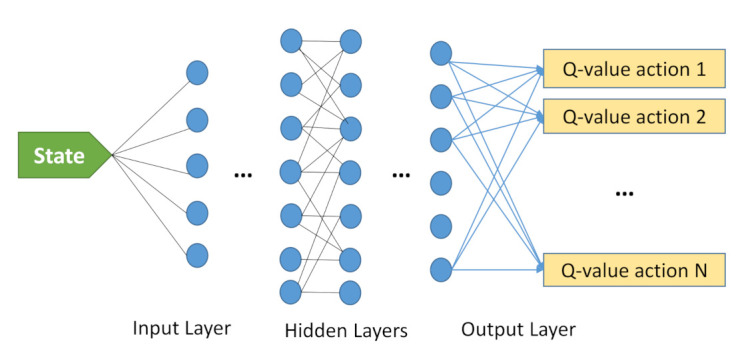
Deep Q-learning model.

Figure 2 represents the Deep Q-learning model to solve a problem. The input is the state information, and the output is a set of estimated Q-values, where our goal is to pick its maximum one. The number of outputs represents the size of action space. One or more hidden layers can exist in a deep Q-learning model. Decaying Epsilon Greedy is another technique that can be employed in DQN to obtain better performance.

## 5. Proposed Model

In the following, we present our Q-learning and Deep Q-learning model proposed to solve the Equation (6).

### 5.1. Proposed Q-Learning Model

In our Q-learning model, since the continuous space is too large and not practical, the target environment is divided into small square cells with equal sizes. The DBS is the agent obviously, who performs the actions while the user moves around the cells.

The components of the model are defined as follows.

State Space: The state represented by $S=[L_{d},L_{u}]$, includes the 2D location of DBS $L_{d}$ and the 2D location of the user $L_{u}$. In this case, the 2D location of the user or the DBS is the center of a cell. Given *C* small cells in the environment, the size of the state space would be C×C.

Action Space: The DBS action space in each state contains eight different moving directions towards a cell that leads to updating the state of the problem. Clearly, the drone cannot perform some of the actions in certain states, due to the environment border. A penalty is then considered for taking these actions.

Reward: The agent needs a reward function that encourages the drone to perform actions reaching a higher performance for the model, as well as avoiding the performance of invalid actions. The main objective is to enhance two-hop communication capacity, therefore, in this model the reward reflects the two-hop communication performance corresponding to the current state of the problem. Note that the state of the problem composed of the location of user and the location of the drone. The DBS selects a direction and moves during the next time step to reach the next state. We choose the two-hop capacity presented in the Equation (5) as a reward to encourage the drone. On the other hand, the agent should be penalized in case of taking certain actions. Hence, the reward function *R* is defined as follows:

(10) $$R\left(s,a\right)=\{\begin{array}{cc} -10, & \;\;\mathrm{if}\;a\notin valid-actions\\ \min(C_{d,u},C_{m,d}) & \mathrm{otherwise}. \end{array}$$

The Q-values are then updated according to the Equation (7), and the learning process repeats until converging to a Q-table. Finally, the drone performs actions following the learned Q-table during its mission. Duration of the missions depends on the battery level of the DBS. Given the equal time steps and constant drone speed, the battery depletes after a particular number of transitions of drones between states.

We will apply the decaying epsilon greedy in our Q-learning model where the epsilon starts with 1, and its value reduces gradually according to the following equation, until reaching to zero.

(11) $$\epsilon =e^{\left(-1*i*decay\_rate\right)},$$

where *i* increases by each learning step.

### 5.2. Deep Q-Learning Model

In the proposed Deep Q-learning model, we use the same action space as the Q-learning model, as a result, the drone can take any of the eight directions at any time step. However, the Q-learning limits on the number of states does not exist in DQN. Therefore, the states composed of the continuous 2D location of the user and the DBS anywhere in the target environment. The same decay epsilon greedy model is also applied in order to create a balance between exploration and exploitation.

Instead of finding Q-values by Q-table, we used three dense layers, with ‘*Tanh*’ activation function. The number of hidden neurons of each layer is 128, 128, and 64, respectively. Obviously, for the last layer of our DQN model, the number of neurons is equal to the size of the action space, with ‘*linear*’ activation function.

Moreover, both experience replay and target network model are applied to our model in order to avoid instability in training. The size of the replay buffer and more details on its implementation are discussed in Section 6. The pseudo-code of DQN is presented by Algorithm 1.
**Algorithm 1** DQN Algorithm1:CreateaReplayBufferB2:Initiaterandomlythelocalnetworkweightsθ3:$\mathrm{Initiate}\;\mathrm{target}\;\mathrm{network}\;\mathrm{weights}\;\theta_{t}\leftarrow \theta$4:**for**eachepisodee**do**5:    Initiatearandomstates6:    **for** eachtransitiont **do**7:        Selectactionaaccordingtoϵ-greedymodel8:        Findthenext-stateandreward9:        StoretheexperienceinbufferB10:        Selectbatch-sizerandomexperiencesfrombufferB11:        CalculateQ(s,a)usinglocalnetworkand12:        thebatchofexperiences13:        $\mathrm{Calculate}\;Q^{\prime }\left(s^{\prime },a\right)\;\mathrm{using}\;\mathrm{target}\;\mathrm{network}\;\mathrm{and}\;$14:        thebatchofnext-states15:        $\mathrm{Calculate}\;\mathrm{loss}\;\mathrm{function}\;\mathrm{using}\;Q^{\prime }\left(s^{\prime },a\right)\;\mathrm{and}\;Q\left(s,a\right)$16:        **if** t%updating-interval==0 **then**17:           $\theta_{t}\leftarrow \theta$18:        **end if**19:    **end for**20:**end for**

## 6. Simulation and Results

To evaluate the performance of the proposed algorithms, a square area with a side length of 240 m, composed of 144 square-cells are considered in our simulation, each cell having a length of 20 m. There is one mobile user in the environment, and one DBS to serve the user. The MBS is located out of the environment at the location of [1000 m, 1000 m].

The user is moving according to the Random Way Point (RWP) model [35] with the speed range of [0.2–5] m/s. The drone located above a cell can move towards any of its neighbor cells by choosing any of eight different directions. However, as mentioned earlier, any invalid movement towards outside of the environment border will be penalized and will keep the drone above its current location. Additionally, we assume that the drone and the macro base station work at different frequencies, and there is no interference between their transmissions. All the parameters regarding the network model are shown in Table 1.

For the Q-learning and DQN model, we have trained the model with different parameters, as listed in Table 2 in order to obtain Q-values in the training process. The agent is trained during 2000 episodes, each of them composed of 1000 transitions. Afterwards, the trained model is evaluated in both Q-learning and DQN model over 500 independent missions of 1000-transitions. Note that the number of transitions reflects the lifetime of a drone. During its lifetime the drone navigates over the environment in order to improve the user performance.

In DQN, an experience replay buffer of size 5000 event is considered and the batch size is 64. Additionally, the parameters of the target network will be updated at each 25 intervals. As mentioned above, the network consists of three dense hidden layers of 128, 128, and 64 neurons, respectively. In the following, we first review the training process of Q-learning and DQN.

**Figure 3:**
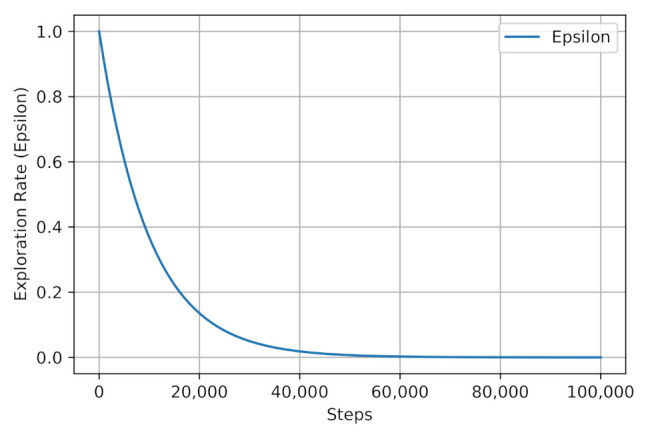
Epsilon value during learning.

Figure 3 shows the epsilon decay strategy where the models select the best action through exploitation first, and gradually the exploration rate increases to benefit the learned Q-values. The same epsilon decay strategy are applied to both Q-learning and DQN models.

**Figure 4:**
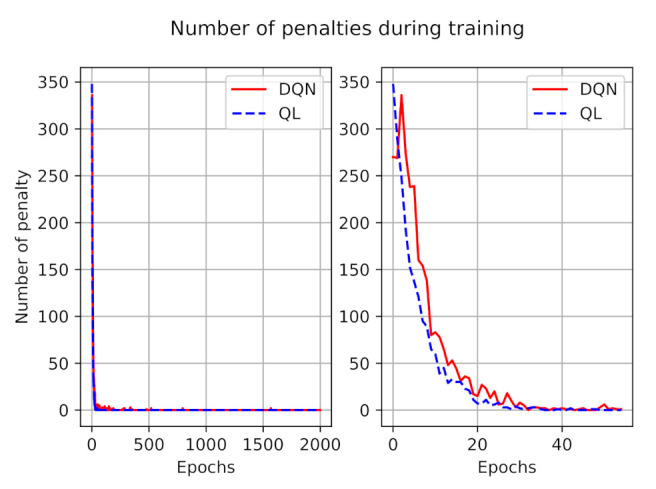
The number of penalties during training.

The number of penalties during the training process for both models is illustrated in Figure 4. As presented in this figure, during the training, at first the agent made mistakes, but over time it learns to avoid invalid actions. The left figure depicted the penalties during all training episodes, and to have a better understanding we zoomed out the number of penalties during the first 50 episodes at the right side. We can observe that, on average, the number of penalties in DQN is larger than in the Q-learning model, however, after a while they both learn from their experiences and the number of penalties tends to zero.

**Figure 5:**
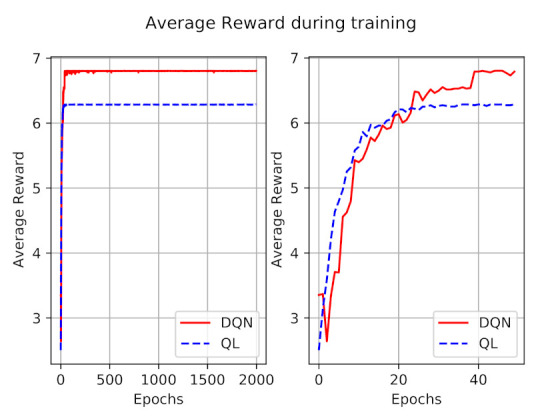
The average reward during the learning episodes.

The average reward during 2000 episodes of training is also shown in Figure 5. Similar to the penalty figure, we have shown the rewards of the first 50 episodes at right to have a better perception. As expected, the reward is increasing during the time, and converges after a while, for both models. At first the obtained reward of the DQN model is less than Q-learning, which totally reflects the impact of a higher number of penalties. After a while, DQN outperforms the Q-learning model in terms of obtained reward.

After learning the Q-table, or approximating Q-values by DQN, we have used the learned values in order to evaluate the behavior of the DBS. To this end, the DBS is operating in 500 different episodes, each including 1000 transactions.

**Figure 6:**
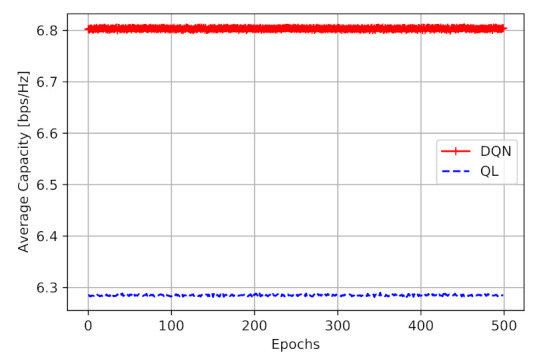
Obtained capacity in evaluation process.

The final goal of the proposed models is to learn DBS to navigate in a way that improves the two-hop capacity. Figure 6 shows the obtained capacity in [bps/Hz]. From this figure, we can perceive that the DQN model has obtained a higher two-hop capacity (average of 6.80 [bps/Hz] ), compared to Q-learning model (average of 6.28 [bps/Hz]). It is also worth mentioning that, during the simulation, the agent makes no mistakes and no penalty, as it is following the learned values. Figure 6 shows that the employment of DQN provides a performance improvement of 8.2% than Q-learning, with a similar action space.

**Figure 7:**
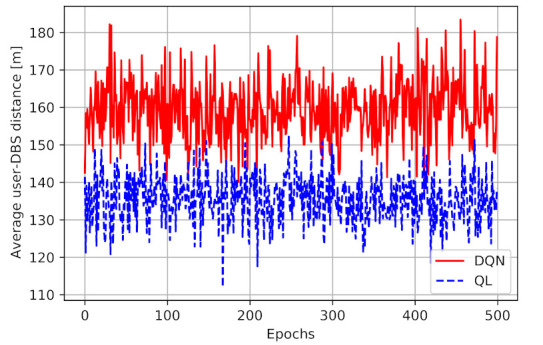
The average distance between the user and the Drone Base Station (DBS) in meter.

Figure 7 presents the average distance between the user and the DBS for both models. As illustrated in this figure, we can observe that the average distance between the user and the DBS in DQN is higher than Q-learning model. The mean distance in a Q-learning model is 135.5 m, while in DQN the number is 159.4m, a 20% increase in distance. First of all, these results show the impact of considering a continuous space in DQN rather than discrete cells in the QL model. Therefore, the DQN model can find better locations for the DBS at which the higher performance can be obtained. Given Equation (5), the obtained performance depends on both the access link and the backhaul link capacity. It means that having a smaller user-DBS distance and higher access link performance does not necessarily end up in higher total performance. The DQN model is managing the navigation better than Q-learning, as it can find the best location at any time for the drone in a way to maximize the two-hop communication capacity.

In order to see the benefit of the reinforcement learning techniques in two-hop joint optimization, we have compared the results with two greedy models. In the one-hop greedy model, at each time the drone moves towards a direction to be closer to the user, to maximize the access link performance. Therefore, at each time step the drone finds a direction that maximizes the access link’s SNR. While in the considered two-hop greedy model, the model tries to maximize the minimum greedy access link and greedy backhaul link. To this end, the access link’s SNR, and the bachhaul link’s performance are calculated for every possible direction. The minimum of these performances are considered as the total performance, and we try to find the direction that maximizes it. Obviously, like the other models, in the greedy models if a selected direction leads the drone out of the border, the drone will stay at its current position. The environment is divided into the discrete cells the same as the Q-learning model.

The greedy models are also evaluated over 500 independent missions of 1000-transitions. The average obtained performance and the user-DBS distance for all models are summarized in Table 3. From this table, we can clearly observe the impact of the two-hop communication on the performance. Additionally, the following points can be detected:

- The proposed DQN model outperforms the single as well as other 2-hop communication models in terms of average network capacity.
- The one-hop greedy model has the minimum user-DBS distance, as this model only focuses on the access link performance and tries to be as close to the user as it can.
- For these simulations, the two-hop model and the Q-learning are generating very similar results. However, it must be noted that after establishing a Q-table for QL model, the computational cost reduces tremendously. In the two-hop greedy model, for any user location all eight different directions must be examined.

**Figure 8:**
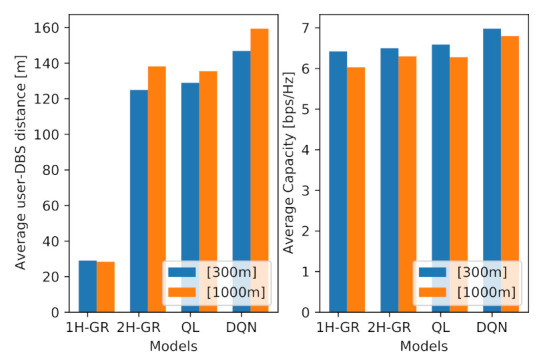
Impact of MBS location on network capacity and user-DBS distance.

Additionally, to see the impact of the location of the MBS on the performance of the proposed models, we have conducted the simulation with an MBS, which is located closer to the target area, at location [300 m, 300 m]. The results are illustrated in Figure 8. From this figure, we can observe the following points:

- The average performance for all models has increased, which is the direct impact of a closer MBS to the environment.
- The average obtained performance in Q-learning and DQN are more than the greedy models, with the average of 6.42, 6.5, 6.59 and 6.98 bps/Hz, respectively.
- Although Q-learning shows slightly better performance than two-hop greedy model (1.3% improvement), their performance still remain very close.
- The average user-DBS distance in one-hop greedy model has not changed significantly, as in this model the drone tries to maximize the access link performance, not taking account the MBS location and the backhaul link.
- As expected, the average user-DBS distance in both QL and DQL models has reduced compared to the further MBS location, which is a direct result of a shorter distance between the DBS and the MBS.

## 7. Conclusions

In this work, we have proposed Q-learning and Deep Q-learning models for a drone base station to serve a mobile user. A two-hop communication scenario is considered, including the access and backhaul links. Our results show that the DBS can learn to navigate autonomously and improve the achievable throughput performance, with DQN outperforms Q-learning. Given the capabilities of DQN, the model can be upgraded to multiple DBSs and users. This upgrade might require a design of a multi-agent reinforcement algorithm model, which is left for our future work. However, for the Q-learning model, extending to a larger environment and multiple users is not practical. Additionally, for real-life deployment, the drone maneuverability limitations and the other dynamics of environments should be further considered.

## Figures and Tables

**Table 1:** Network and Communications parameters.

Parameters	Value
Drone Height	20 m
MBS Height	30 m
Edge Length of a Square Cell	20 m
Are size	240 m
Drone Working Frequency	2 GHz
MBS Working Frequency	1.8 GHz
Drone Transmission Power	30 dBm
MBS Transmission Power	32 dBm
Noise Figure	9 dB
Thermal Noise Density	−174 dBm/Hz
Transmitter’s/receiver’s antenna gain	1

**Table 2:** Q-learning and DQN training parameters.

Parameters	Value
Learning rate (α)	0.01
Discount factor (λ)	0.9
Epsilon-start	1
Epsilon-Decay-rate	0.0001
Training Episodes	2000
Replay Buffer Size	5000 evens
Batch Size	64

**Table 3:** Comparing the models for a Macro Base Station (MBS) located at [1000 m, 1000 m].

**Model**	1Hop Greedy	2Hop Greedy	QL	DQN
**Performance [bps/Hz]**	6.03	6.3	6.28	6.80
**User-DBS Distance [m]**	28.4	138.2	135.5	159.4

## Data Availability

Not applicable.
